# Editorial: Innovative approaches and molecular mechanisms in cardiovascular pharmacology

**DOI:** 10.3389/fphar.2026.1857987

**Published:** 2026-06-15

**Authors:** Maria Luiza S. Silva, Cristal J. Toghi, Carlos Alan C. Dias-Junior

**Affiliations:** Department of Biophysics and Pharmacology, Institute of Biosciences, São Paulo State University, São Paulo, Brazil

**Keywords:** cardiovascular, mitochondrial dysfunction, molecular mechanisms, peripheral arteries, pharmacology

Cardiovascular diseases (CVDs) are a leading cause of death globally, posing significant challenges to healthcare systems and affecting millions of lives. Despite substantial advances in the understanding and treatment of CVDs, there remains an urgent need for innovative therapeutic strategies and a deeper comprehension of the complex molecular mechanisms underlying these conditions. By exploring novel pharmacological interventions and elucidating their molecular targets, we can pave the way for more effective treatments and improved patient outcomes.

This Research Topic aimed to gather cutting-edge research and comprehensive reviews that focus on innovative pharmacological approaches and the molecular mechanisms involved in the treatment and management of cardiovascular diseases. The Research Topic sought to bridge the gap between basic science and clinical application, fostering interdisciplinary collaboration and encouraging the integration of advanced technologies and methodologies.

This Research Topic received several manuscripts that focused on, but are not limited to, the following aspects:

- Drug Discovery - Pharmacological Interventions: Research on new or repurposed drugs targeting cardiovascular diseases, emphasizing efficacy and safety.
- Molecular Mechanisms: Studies unraveling the molecular pathways and targets involved in CVDs, including signaling pathways and genetic factors.
- Biomarkers and Diagnostics: Identification of novel biomarkers for early detection and diagnosis of cardiovascular conditions.
- Therapeutic Strategies: Exploration of emerging therapies such as gene therapy, RNA-based treatments, and regenerative medicine.
- Cardiotoxicity: Insights into the cardiotoxic effects of non-cardiovascular drugs, including mechanisms and mitigation strategies.
- Technological Innovations: Application of novel technologies like bioinformatics and AI in cardiovascular research.

Articles demonstrated and highlighted:

- The crosstalk between mitochondrial dysfunction and fatty acid metabolism in heart failure: Mechanisms and therapeutic strategies (Wang et al.);
- Potential Prognostic Biomarkers in Restenosis of Peripheral Arteries Identified Through Comprehensive ceRNA Network Analysis (Miao et al.);
- Bitter Taste Receptor Agonists Induce Vasorelaxation in Porcine Coronary Arteries (Tsai et al.);
- The Role of Exosomes Derived from Macrophages in the Progression of Cardiovascular Disease through Physiological Processes (Qi et al.);
- Effect of Sodium-Glucose Co-Transporter 2 Inhibitors on Coronary Microcirculation (Chen et al.).

Summarizing the contributing articles, this Research Topic aimed to advance the field of cardiovascular pharmacology, enhanced therapeutic approaches, and ultimately improved patient outcomes.

